# Auditory cognition lab: a music therapy and speech-language pathology co-treatment for military-connected populations with auditory and cognitive impairment

**DOI:** 10.3389/fneur.2025.1642056

**Published:** 2025-09-01

**Authors:** Danielle Vetro-Kalseth, Rebecca Vaudreuil, Heather Morrison, Kathleen Howland, Hannah Bronson

**Affiliations:** ^1^Henry M. Jackson Foundation for the Advancement of Military Medicine, Inc. in support of Creative Forces: NEA Military Healing Arts Network and Center for Rehabilitation Sciences Research at the Uniformed Services University of the Health Sciences, Bethesda, MD, United States; ^2^673rd Medical Group, Joint-Base Elmendorf Richardson, Anchorage, AK, United States; ^3^Music Therapy Department, Berklee College of Music, Boston, MA, United States; ^4^Polytrauma System of Care, VA Palo Alto Health Care System, Palo Alto, CA, United States

**Keywords:** music therapy, speech language pathology, auditory processing, cognition, military, traumatic brain injury, post-traumatic stress disorder

## Abstract

Military personnel are particularly at risk for auditory processing difficulties as their training, occupational, and combat experiences increase the likelihood of long-term damage to the auditory system as well as negatively impact physical, psychological, cognitive, and sensory functioning. This article introduces a music therapy and speech-language pathology co-treatment program, Auditory Cognition Lab (ACL), that treats auditory and cognitive deficits in military-connected populations (service members, veterans) with traumatic brain injury (TBI). ACL addresses auditory discrimination, temporal and binaural processing, and trains compensatory strategies related to auditory processing, cognition, and hearing function. ACL has been clinically implemented at multiple military treatment facilities across the United States. Preliminary outcomes have demonstrated improvement in processing speed, auditory perception, active listening, expressive and receptive language, memory encoding and retrieval, attention at varying levels (sustained, divided, alternating), and self-efficacy. Further research is warranted to learn more about the benefit of this innovative co-treatment program for military-connected individuals with auditory processing deficits and TBI. This paper provides a theoretical framework, comprehensive description and critical reflection of this intervention, and outlines a research strategy for a current feasibility and acceptability study.

## Introduction

Processing sound is an essential neurological function for interpreting external stimuli, orienting to environments, and engaging in the world. The brain’s auditory system is complex, and structural damage to these areas can impact functioning in social, emotional, physical, and cognitive domains (1, 2, 111). An auditory scaffolding hypothesis posits that auditory processing is the foundation of cognition during childhood and changes in auditory function (hearing loss, distortions) can negatively affect cognitive development (3). Military personnel are at higher risk for auditory processing dysfunction (4, 5). Their training, occupational, and combat experiences include tactics such as breaching, firing of heavy artillery (shoulder mounted weapons), contact with improvised explosive devices creating low-level blast exposure, and interaction with kerosene-based jet fuel. These exposures increase the likelihood of long-term damage to the auditory system as well as other physical, psychological, cognitive, and sensory disturbances (5). In addition to self-report, objective physiological and behavioral measures indicate that auditory processing impairment often accompanies concussion even when there is no evident brain damage detected by imaging technology (6, 7).

Military acquired auditory processing dysfunction often accompanies complex physical and psychological injuries (4, 5). Recent military conflicts have resulted in high rates of ‘signature wounds of war,’ namely post-traumatic stress disorder (PTSD) and traumatic brain injury (TBI). The prevalence of TBI, PTSD, and related sequelae has prompted a need for interdisciplinary rehabilitative treatment approaches that address the range of symptoms accompanying these diagnoses (8). Additionally, the Defense Health Agency’s (DHA) Strategic Plan (2023–2028) emphasizes a modernized approach to healthcare, which aims to improve well-being and maintain readiness through scaling innovative, evidence-based, and patient-centered care (9). For this reason, providers in military treatment facilities seek novel, integrative, and interdisciplinary opportunities to address the complex needs and overlapping symptomatology that accompanies TBI and PTSD, ultimately returning service members to full duty. Furthermore, treatments that demonstrate efficacy and are replicable within healthcare systems that support both the active-duty service members and veterans, the DHA and Veterans Health Administration (VHA), respectively, are warranted.

Board-certified music therapists (MT-BCs) and speech-language pathologists (SLPs) work as part of interdisciplinary programming in military healthcare providing rehabilitation across the spectrum of audition including challenges with music and speech (10). MT-BCs assess and treat issues related to emotional, physical, cognitive, and social functioning through evidence-informed music therapy interventions (11). SLPs operate within multidisciplinary teams in collaboration with other disciplines (audiology, neuropsychology, neurology) to screen for, evaluate, and treat cognitive and auditory processing disorders. Assessment of cognitive strengths and weaknesses can be used to inform auditory processing training, which often involves best available practices including skill-based training on areas of weakness and compensatory strategies (12).

Continued efforts leverage the unique and important contributions of MT-BCs and SLPs within interdisciplinary care models in military medicine. MT-BCs and SLPs working collaboratively in these settings across the United States (U.S.) have developed and clinically implemented a co-treatment program, Auditory Cognition Lab (ACL), which is a series of sessions focused on rehabilitating auditory and cognitive functioning through clinical intervention and skills-based training. ACL was established in 2016 by MT-BCs and SLPs working at a DOD clinic that specializes in treating TBI and has since been innovated at multiple DOD sites including the mild TBI (mTBI) clinic at Joint Base Elmendorf-Richardson in Anchorage, Alaska. Across sites, ACL is co-facilitated by MT-BCs and SLPs as part of intensive and longitudinal outpatient programming. While the interventions contained within ACL are well accepted and empirically validated in both music therapy and speech language pathology professions, the unique combination of these approaches may augment the efficacy of either set of interventions alone (13). Preliminary development has resulted in varying permutations of ACL, adapted by SLPs and MT-BCs within the DHA based on their unique clinical environments and program models. Observational data collected through initial program development has shown promise. These include clinician developed qualitative surveys (Boxes 1, 2) and unpublished findings from standardized self-report symptom measures such as the Hearing Handicap Inventory for Adults (14), Communication Confidence Profile (15), and Self-Efficacy for Symptom Management Scale (16). Additionally, performance-based assessments related to auditory processing including the Quick Speech in Noise (17) and SCAN-3 Tests for Auditory Processing Disorder (18) have also indicated improvement in these areas. While initial clinical findings demonstrate promise, further standardization and manualization of ACL will allow for replication and clinical trials that examine its efficacy. Developing standardized treatment interventions and clinical protocols that address the unique needs of military-connected (service members, veterans) populations is essential to assisting in military readiness and reintegration. This paper introduces and describes the development of the ACL co-treatment program that utilizes an integrated approach to auditory and cognitive rehabilitation of military-connected patients in both DHA and VHA healthcare, and outlines a research strategy for a multi-site feasibility and acceptability study that is currently underway across these systems.

BOX 1 **ACL participant feedback (1)**

*“This program has helped me feel more confident in my ability to make the adjustments I need in order to hear and understand.”—ACL Participant Feedback*

BOX 2 **ACL participant feedback (2)**

*“In this program I learned that having processing difficulties is not something to be embarrassed about and I learned ways to overcome the difficulties. [ACL] taught me to push myself outside of my comfort zone. It has helped me to be better at noticing things, including what I can do in any given situation to be more successful.”—ACL Participant Feedback*

## Traumatic brain injury and co-occurring conditions

According to the U.S. Centers for Disease Control and Prevention (CDC) (19), TBI can be caused by external forces such as a bump or blow to the head and internal penetrating injuries that affect brain functioning. The CDC recognizes three main types of TBI including mTBI also referred to as “concussion,” moderate TBI, and severe TBI, which are primary concerns in military healthcare (9). Since 2000, there have been over 505,896 reported cases of TBI in the military, 81.9% of which are classified as mTBI (9, 105). In addition, elevated biomarkers associated with TBI have been identified in service members with chronic low-level blast exposure (20, 106). Military service members chronically exposed to repeated blasts throughout their career may experience a cumulative injury effect resulting in long-term neurological sequelae (21). Biomechanistic studies suggest associations between cumulative blast exposure and changes in structure and brain function in the frontal lobe, subsequently impacting quality of life (22). Longitudinal studies in blast-exposed populations also show functional disruption to central and peripheral sensory systems, potentially signaling an interaction leading to changes in auditory processing abilities (23).

Several studies (4, 5, 24–30, 109) have linked auditory processing deficits to mTBI and/or blast exposure in military-connected individuals. When compared to those without mTBI and/or blast exposure, service members with a blast-related injury are twice as likely to have peripheral hearing loss, underscoring the auditory system’s vulnerability to these exposures (4). Sensorineural hearing loss and other auditory disruptions such as tinnitus can be co-occurring with central auditory processing deficits, which may complicate diagnostic clarification. In a study examining auditory and cognitive performance deficits, participants with mTBI reported higher percentage of tinnitus (66% vs. 16% in control group) and 88% reported moderate to very large impact of their injury on their listening skills. When excluding participants with insufficient effort, significant differences in auditory performance between the control group and the mTBI group were observed (31). Hoover et al. (32) also found that 84.6% of study participants with mTBI (*n* = 13) endorsed difficulty hearing speech in the presence of background noise compared to 0% reporting difficulty hearing speech in a quiet setting. Those who endorsed difficulties were more likely to demonstrate lower speech-in-noise scores on objective measures, suggesting the need for comprehensive audiological evaluation including both pure-tone audiometry to assess for sensorineural hearing loss as well as speech-in-noise tasks (32). Additionally, high-intensity blast exposure resulted in lower performance on central auditory processing tests in 83 out of 1,000 military personnel (24). Cortical functions rely on sensory and somatic input from the nervous system for higher order processing involved in memory, language, and attention (33). Therefore, it is not surprising that related studies within the VHA indicate that between 62 and 92.5% of veterans with mTBI self-report difficulties with auditory functioning including central auditory processing disorder (CAPD) (27, 30, 34).

### Central auditory processing disorder

The American Speech-Language-Hearing Association (ASHA) defines CAPD as “deficits in the neural processing of auditory information in the central auditory nervous system not due to higher order language or cognitive factors” (35) (para 4). Individuals with CAPD may demonstrate impaired functioning in areas of auditory discrimination, pattern recognition, and auditory performance in the presence of competing sounds and/or degraded acoustic signals (36). This is due to hearing loss, auditory distortions, tinnitus, or a combination of these factors. Such auditory deficits are more prevalent in military-connected individuals compared to civilians (37, 38). This is largely related to the uniqueness of military occupational hazards such as blast exposure (24, 28), persistent subjection to destructive noise (39, 40), exposure to chemicals (41, 42), and neurotrauma including mTBI (5, 30). The prevalence of auditory processing deficits is hypothesized in a study among service members exposed to blast within 1 year, where 74% performed abnormally on at least one test of central auditory functioning, and 44% performed abnormally on two or more tests, compared with 24 and 10% of controls, respectively (24). Tepe et al. (5) discusses challenges in accurately quantifying the prevalence of auditory processing disorders including lack of appropriate screening, service member’s stigma of hearing loss, misinterpretation of available sensory input, and limited referrals for diagnostic confirmation. Furthermore, it is estimated that 5.7% of service members are at an elevated risk for CAPD, which may be undetected if they meet fitness for duty standards on audiometric exams including those who demonstrate normal to near normal hearing thresholds (5).

### Post-traumatic stress disorder

In addition to disruption to auditory processing systems, mTBI and low-level blast exposure in military service members often is accompanied by psychological trauma, subsequently interfering with overlapping neurological systems involved in attention and concentration (43). Exposure to trauma can result in long-term alterations to emotional and cognitive functioning due to maladaptive neuroplasticity (44). Traumatic stress reactions may manifest as a psychiatric condition such as PTSD if symptoms persist for a determined duration and meet specific criteria such as experiencing intrusion symptoms, avoidance, hyperarousal, and negative affect (45). The U.S. Department of Veterans Affairs (VA) describes PTSD as a mental health condition that may develop after a directly experienced, witnessed, or threatened traumatic event such as warfare, physical, and/or sexual assault (46). The VA reports that the prevalence of PTSD for Operation Enduring Freedom and Operation Iraqi Freedom Veterans occur at the rate of 15% per year and that 29% of all U.S. Veterans from these service eras will be diagnosed at some point in their lives (46, 108). Symptoms of PTSD involve alterations to cognitive processes such as memory, attention, planning, and problem solving, thus underscoring the detriment of negative emotionality on cognitive functioning (47). Reavis et al. (4) posits that the interaction between blast exposure and resulting hearing sequelae is potentially mediated by PTSD. This may be due to changes in neurotransmitter activity such as serotonergic concentration, which subsequently increases hypersensitivity to sound while reducing effective sensory gating as modulated by the brainstem (48). The resulting decrease in voluntary control of attention can impact higher-level cognitive processes such as auditory working memory, executive function, and social–emotional learning (49). The aforementioned unique occupational exposures resulting in comorbid neurological (CAPD, TBI) and psychological (PTSD) diagnoses and symptom overlap warrant an interdisciplinary treatment approach. Thus, further research is needed to identify effective treatments that optimize rehabilitation while addressing overlapping goal areas (30).

## Music therapy to address auditory and cognitive functioning

Military-connected individuals with TBI may experience cognitive, functional, and health impairments that adversely affect their ability to function during and after military service (50). There is growing evidence that music therapy is a viable treatment option for persons with neurologic injury including concussion (51), auditory processing related deficits (52), and behavioral health issues (53). MT-BCs are healthcare professionals who are trained to effectively integrate into interdisciplinary treatment. Many MT-BCs who treat cognitive conditions specialize in Neurologic Music Therapy (NMT) (Table 1), which is a practice that uses standardized techniques to address cognitive, communication, and sensorimotor disorders by pairing music therapy interventions with underpinnings of neurobiology (10, 54–57). Music engages neural networks related to auditory processing, which may serve to increase neuroplasticity, support cognitive priming, and promote patient motivation in rehabilitation (58).

**Table 1:** Neurologic Music Therapy techniques used in ACL.

NMT technique	Description	References
Auditory Perception Training (APT)	Focuses on auditory perception and sensory integration using musical exercises that facilitate the identification and discrimination of music elements.	Weihing (72)
Musical Attention Control Training (MACT)	Provides structured activities to focus, maintain, and switch attention based on musical cues.	van Alphen et al. (103)
Musical Executive Function Training (MEFT)	Provides musical activities that engage decision making, organization, problem solving, and reasoning skills.	Rodriguez-Gomez and Talero-Gutiérrez (104)

Active and receptive music therapy interventions engage different levels of neural activity. For example, studies have shown that listening to music activates different neural processes when compared to active music making using an instrument or voice (59). However, even at a passive level of music listening, multiple studies report the alleviation of perceived anxiety and physiological markers of stress during painful medical procedures (60–63). Therefore, music therapy interventions employed in ACL seek to target levels of neural functioning by providing predictable and salient stimuli that may regulate healthy brain functioning.

Music stimulates widely distributed neural networks beyond those typically engaged in general cognitive, language, and motor processes (64, 65). In a single-blind crossover randomized control trial (*n* = 20), individuals with TBI of at least moderate severity receiving 20 individual NMT sessions demonstrated increased connective white matter of the right frontal dorsal, projection pathways, and corpus callosum via increased quantitative anisotropy compared to standard care (51). Many neuroimaging studies have found that music perception activates the temporal, parietal, frontal, cerebellum, and subcortical regions of the brain. These areas control information processing speed, attention, and memory functions (working, episodic, short and long term), reasoning, motor functions, processing of music semantics and syntax, and creativity (58, 66, 67). The activation of these neural networks can be leveraged to improve functioning within and across areas of cognition (68, 69).

## Speech language pathology to address auditory and cognitive functioning

SLPs provide comprehensive evaluation and treatment of multiple areas including cognition, speech, and language. Thorough assessment may identify other diagnoses that present with similar characteristics to CAPD including speech-language dysfunction, cognitive-communication issues, and/or attention-deficit/hyperactivity disorder (ADHD). Comprehensive SLP assessment may inform the need for an audiology referral that can confirm a CAPD diagnosis (35).

After diagnostic confirmation of an auditory processing disorder by an audiologist, SLPs’ input contributes to the translation of audiological findings to functional contexts by developing appropriate treatment strategies and recommendations (35, 70, 71). Patient-centered treatment plans focus on improving auditory processing through direct skill training beginning with areas most negatively impacted. These may include speech in the presence of background noise/binaural separation, gap detection/temporal resolution, and/or phoneme discrimination. Training in these areas can improve skills, promote generalization, and reduce functional deficits by focusing treatment on learning strategies and slowly increasing exercise complexity (72, 107). Computer-based training programs may be utilized to allow for continued skill reinforcement at home and to increase repetition of practice. SLPs provide treatment aligned with the recommended dosages of duration and length of treatment. However, the treatment frequency rates that are considered a standard of care in many well-recognized auditory training protocols is often unattainable due to limited patient access and scheduling constraints (5). Therefore, SLPs may recommend computer-based training programs that allow for continued practice of specific skills (speech-in-noise, competing sounds, etc.) and can be completed outside the clinic at higher frequency. SLPs may also recommend specific strategies for managing auditory processing difficulties.

In addition to direct skill training, SLPs provide recommendations for modifications of the listening environment, speaker adaptations (pausing, emphasizing key words, sitting closer to the speech signal, etc.), and provide potential supports such as pairing verbal presentation with handouts or visual aids. SLP interventions include active listening and/or attention training to enhance and improve efficiency of auditory perception by targeting attentiveness to a signal. Assistive technology devices such as low-gain hearing aids or frequency modulation systems for hearing can help to eliminate or attenuate competing sounds (5). Environmental modifications and compensatory strategy training can help to reduce the negative impacts of CAPD by enhancing performance in functional, everyday contexts.

## Clinical and research initiatives

Development of clinical and research initiatives for military-connected populations with complex and co-occurring conditions is collaboratively supported by multiple federal agencies and academic institutions. The National Institutes of Health (NIH) in partnership with the National Endowment for the Arts (NEA), the Kennedy Center, and University of California San Francisco created the Sound Health Network that investigated relationships among music and neural mechanisms (73). Additionally, the NEA established Creative Forces ®: NEA Military Healing Arts Network, an initiative in partnership with the U.S. Departments of Defense (DOD) and VA. Creative Forces (CF) seeks to improve the health, well-being, and quality of life for service members and veterans exposed to trauma and their families and caregivers (74). This is accomplished by demonstrating the value and impact of the arts to include creative arts therapies (art therapy, music therapy, dance/movement therapy) with military-connected populations exposed to trauma. Creative arts therapies are healthcare professions that use arts-based clinical processes and protocols to address physiological, psychological, and social well-being of diverse individuals and communities (75).

Clinically, CF supports creative art therapies as a standard of care at 12 DOD and VA sites, as well as a telehealth program (50, 76, 77). Preliminary CF evaluation data and research indicates that creative arts therapies can: improve awareness and reduce symptoms associated with PTSD/TBI (50, 78), increase strengths-based rehabilitation (79), enable recovery from traumatic experiences through verbal processing (80), reduce isolation and stigma (78), increase co-treatment opportunities (81), and improve communication with family, peers, and providers (10, 57). The strategic placement of creative arts therapists within interdisciplinary care teams that treat service members and veterans with complex injuries and illnesses fosters rich opportunities for collaborative clinical research. CF aims to contribute to emerging science by identifying key priority areas for study that optimize sustainable clinical outcomes through evidence-based practice for military service members and veterans (82).

In order to advance the understanding of creative arts therapies with military-connected populations, the National Defense Authorization Act for Fiscal Year 2023 awarded funding to Uniformed Services University and the Center for Deployment Psychology to further empirical research on the effectiveness of creative arts therapies for TBI. Primary objectives of this effort include to (1) evaluate the efficacy of creative arts therapies for the treatment of TBI sequelae and co-occurring conditions (PTSD, chronic pain, sleep disturbances), (2) develop and/or systematize creative arts therapies interventions, and (3) develop and/or evaluate psychometrically sound and/or empirically validated assessment tools for outcomes or processes associated with creative arts therapies (83). A three-year feasibility and manualization study of ACL was reviewed for scientific merit and funding recommendations by the NIH and meets objectives (1) and (2) by both examining outcomes and trends in symptom reduction of TBI sequelae through standardizing ACL for implementation at one DOD site and one VHA site.

## Methods

### Auditory cognition lab co-treatment program and clinical protocol

DeGraba et al. (84) report on the efficacy and efficiency of interdisciplinary care models to motivate patient participation in rehabilitation. Music therapy and speech-language pathology are complementary and effective in addressing neurologic functioning, which allows for co-treatment and innovative approaches to care (10, 13). While these two disciplines can be facilitated as stand-alone treatments, co-treatment efforts between MT-BCs and SLPs may increase patient outcomes (10). In addition, both disciplines are auditory practitioners and interventions within both professions address the auditory spectrum of neurologically salient information through multimodal approaches. Therefore, quantifying the augmentative impact of music therapy and speech-language pathology co-treatment for military-connected populations is necessary.

ACL is a set of clinical interventions derived from music therapy and speech-language pathology organized as a group co-treatment for military-connected patients with mTBI who experience cognitive and auditory processing issues such as sensorineural hearing loss, CAPD, and/or tinnitus. ACL is composed of six 60-min group sessions that address auditory discrimination, temporal and binaural processing, and foundations of speech, language, and audition combined with elements of music such as pitch, tempo, rhythm, and volume. ACL is designed to improve auditory processing and cognition systems through integrated musical and communication exercises that incorporate both bottom-up training by addressing specific skills (auditory discrimination, temporal processing), top-down training of compensatory strategies (theme identification, note-taking), and making environmental modifications such as minimizing competing sounds (85).

ACL aims to improve auditory perception, binaural processing, expressive and receptive language, memory encoding and retrieval, varying levels of attention (sustained, selective, alternating), and information processing and comprehension. ACL facilitates auditory training (speech-in-noise, rapid speech, etc.) and provides education on compensatory approaches including auditory processing strategies, active listening skills, and metacognitive awareness. MT-BCs and SLPs co-facilitate interventions and share knowledge from their respective fields that help patients generalize information and skills from group sessions to their daily routines. Throughout the ACL treatment program, patients attend to progressively longer and more complex auditory stimuli. They are prompted to use working memory and decision making to determine the key words in a passage or summarize material heard, apply strategies for emotional regulation during stressful listening situations, determine when to apply environmental modifications and compensatory strategies, and implement active listening skills. Patients also learn strategies that strengthen neuroplasticity and auditory processing, which may increase higher-level cognitive performance including executive functioning. Clinicians provide opportunities to practice self-monitoring and self-assessment throughout treatment and patients are encouraged to utilize the strategies in different environments and listening situations. Skills are then ideally carried over and implemented in external settings with a wider range of sensory stimuli. Individual outcomes within the group are measured by response sheets that correspond to each exercise, which highlight areas that may require more support and are also used to track treatment progress throughout the six sessions. Table 2 demonstrates an outline of the target areas, interventions, and exercises included in ACL.

**Table 2:** ACL session outline.

Session	Target areas	Interventions, techniques, and exercises
1	Auditory Working Memory, Gap Detection	MT Intervention: Active music making/rhythm exercises NMT Technique: MACT SLP Intervention: Psychoeducation, compensatory strategy training (attention, environmental) SLP Exercises: Training attention strategies
2	Auditory Vigilance	MT Intervention: Pitch identification NMT Technique: APT, MACT SLP Intervention: Attention training, active listening, coping strategies SLP Exercises: Alert to sounds over time, identify pitch rise/fall and emphasis in speech
3	Auditory Discrimination	MT Intervention: Active music listening NMT Technique: APT, MEFT SLP Intervention: Attention and executive function training, auditory discrimination training, effective communication strategies SLP Exercises: Active listening, minimal pairs
4	Intensity Detection, Gap Detection	MT Intervention: Active music making, Intentional listening NMT Technique: APT, MACT SLP Intervention: Attention training, active listening SLP Exercises: Sound identification and discrimination, gap detection
5	Competing Sounds, Minimal Pairs	MT Intervention: Intentional listening NMT Technique: MACT, MEFT SLP Intervention: Environmental modification, self-advocacy training/modeling repair strategies, attention training SLP Exercises: Auditory comprehension, attention training, minimal pairs
6	Following Directions, Compensatory Strategy Training	MT Intervention: Active music making NMT Technique: MEFT SLP Intervention: Following simple/complex directions SLP Exercises: Compensatory strategies, effective communication, following multi-step auditory instructions

NMT Techniques: APT, Auditory Perception Training; MACT, Music Attention Control Training; MEFT, Music Executive Function Training.

#### Auditory discrimination

Pitch discrimination exercises train patients to distinguish between high and low musical notes, which assists them in determining variation in sounds (86). Words that differ by a single sound, have a higher or lower pitch, or differ in volume can be difficult to distinguish in conversation. Subtle pitch, volume, and overall sound changes can alter meaning and disrupt verbal communication (87).

#### Temporal processing

Gap detection and echoic memory exercises train patients to interpret pauses in communication to better express and receive informational content (88, 89). Rhythmic exercises are facilitated to increase understanding of temporal distinctions that occur in speech patterns and train the ability to perceive messages presented with rapid speech (56).

#### Binaural processing

Binaural processing allows for attending to important auditory information and filtering out insignificant information (112). Exercises using competing sounds train participants on strategies that they can employ when encountering complex listening environments and assists with understanding conversational content. This is especially helpful for listeners with hearing complications (hearing loss, tinnitus) and/or when voices are altered (sociolinguistics, talking on the phone). Auditory vigilance exercises train the ability to maintain concentrated attention over periods of time (90).

### ACL session descriptions

Session one introduces the treatment, concepts of compensation and restoration in cognitive rehabilitation, and the cognitive pyramid (110). Informational resources (handouts, worksheets) provide a heuristic conceptualization of the relationships between neuropsychological and cognitive constructs to support patients’ understanding of these core functions related to the treatment. Exercises train echoic/auditory memory reinforced by rhythmic stimuli to improve attention over time. For example, patients engage in group drumming by playing together in rhythm on one beat – the ‘downbeat’. Once rhythmic synchronization is accomplished, patients ‘pass the beat’ by taking turns playing the next beat on their drum in successive rhythm to the established downbeat. Layers of complexity can be added by identifying a cue (verbal, sound) that prompts the patients to change playing patterns. Gap detection training supports echoic memory by hearing two similar sounds and determining the difference between beats (one sound) and flams (two sounds played close together). Patients are positioned facing away from the music therapist who plays either a beat or flam and use auditory discrimination to identify which sound was played. All sessions conclude with group processing of the experiences and resourcing for patients to use outside of the clinical session, generalizing trained skills to real-life application.

Session two begins with a review of content from session one and a group discussion regarding compensatory strategies patients may have utilized between sessions. All sessions begin this way as treatment progresses. Exercises presented in the second session include active listening, responding to sounds, and sustaining concentrated and alternating attention to address auditory vigilance. For example, patients are asked to listen to minute-long audio recordings and count how many times they hear certain sounds. This is done with a single sound (a text message alert), two sounds (higher and lower pitched), and environmental sounds (a baby crying while dogs are barking). Pitch discrimination exercises are helpful for communication, as different pitches can relay certain aspects pertaining to conversation and comprehension (91). MT-BCs play two pitches successively on various instruments (piano, guitar, voice) and patients determine if the second pitch is higher or lower than the first. The concept of prosody (vocal intonation) is introduced through exercises in which patients are trained to distinguish between questions and statements by determining if pitch is rising, falling, or staying the same in a spoken story and a live song.

Session three addresses time compressed/rapid speech. Patients identify keywords and then listen to information delivered at varied speeds. The complexity gradually increases from slow to moderate and fast speeds providing scaffolded challenges to auditory processing. Building upon previous tasks, more information is delivered in story form and as full songs in which patients identify overall themes using keywords and comprehension questions. Minimal pair exercises promote active listening to detect variance between sounds in word pairings that differ by only one sound (initial and final consonants) and have different meanings (92). These exercises train compensatory strategies for everyday life scenarios.

Session four trains intensity and gap detection using drumming, singing, and spoken word. Intensity detection includes speech and music exercises in which patients determine the intensity (volume) of sounds given in single- and two-note and in single- and two-word intensity. Patients are asked to match the intensity through playing it on a drum and repeating spoken words. Gap detection addresses the perception of space between multiple sounds through music and speech-based active listening exercises. Patients are asked to detect gaps between words in a spoken story and in sung lyrics of a song.

Session five addresses competing sounds, which trains the ability to focus on various types of auditory stimuli. It requires patients to shift attention and interpret spoken words presented through speech and sung lyrics. Like previous sessions, tasks increase in complexity from listening to lyrics with no competing sound(s) to alternating attention between spoken words and sung lyrics with live instrumental accompaniment. Between each exercise, patients answer questions related to comprehension, find missing words, and identify key themes. For example, patients fill in missing lyrics to a song played live by the MT-BC while the SLP simultaneously narrates a story. Another task involves patients alternating their attention from a spoken story to a song played live and identifying themes in both.

Session six focuses on following directions to increasingly complex tasks using compensatory strategies. Patients follow verbal and pictorial directions to complete a simple paper folding task while implementing compensatory strategies. Patients engage in active music making by retaining and recalling information provided via verbal instruction by the MT-BC. Lastly, patients engage in group music making on a variety of instruments to a three-chord song. These exercises train patients’ ability to sustain attention, comprehend and memorize content, while identifying their ideal learning styles for following directions. This is accomplished through live music experiences, utilization of applied strategies, and group discussion.

### Study design

This study employs a non-randomized, mixed methods design to assess the feasibility and acceptability of ACL in military-connected populations with TBI and military acquired auditory processing dysfunction. Study objectives include (1) assessing the feasibility and acceptability of ACL in military and veteran healthcare settings and (2) exploring trends in auditory functioning, post-concussive symptoms, anxiety, depression, and perceived stress following ACL participation. Feasibility and acceptability will be assessed through provider-level focus groups (SLPs and MTs) aimed at reviewing the ACL program prior to clinical implementation. Feedback gleaned from these focus groups will inform standardization, manualization, and refinement of content within ACL from its existing versions.

Acceptability from clinicians facilitating ACL as part of the study will be assessed through a survey designed to capture successful implementation as well as any critical barriers. Trends in auditory functioning in participants receiving ACL will be assessed by the Speech and Spatial Qualities of Hearing Scale (SSQ-12), the SCAN-3 Tests for Auditory Processing Disorders in Adults, and the Tinnitus Handicap Inventory (THI) (18, 93, 94). Changes in post-concussive symptom reporting will be measured by the Neurobehavioral Symptom Inventory (NSI) (95). Heart Rate Variability (HRV) is a commonly used biomarker measured by wearable devices; higher HRV signals a more adaptable autonomic nervous system as well as positive emotional health (113). HRV will be measured via a wearable biofeedback device that can be worn during participation in ACL sessions. Changes in overall subjective well-being and negative symptoms related to psychological health will be measured by the Satisfaction with Life Scale (SWLS), Patient Reported Outcomes Measurement Information System (PROMIS) Emotional Distress Anxiety and Depression Short Forms, NIH Perceived Stress Scale V3.0, and the Self-Efficacy for Symptom Management Scale of TBI (SESM) (16, 96–99). A Music Therapy Symptom Assessment short form was developed by music therapists within the CF network for pre- and post-session administration and includes a Likert scale (0–10) rating common symptoms reported by service members receiving care in interdisciplinary TBI clinics (pain, physical tension, trouble focusing, sadness, anger) (81). Qualitative data will be collected through semi-structured interviews with participants after completing the ACL intervention protocol.

Six 60-min sessions, as outlined above, will be administered either once a week for 6 weeks or twice a week for 3 weeks within either outpatient or residential treatment based on site-specific clinical programming. Outcome measures will be collected at various time stamps including upon enrollment as baseline, pre- and post-session to capture within session data, mid-protocol after the third session, and 1 to 3 weeks post-intervention. Table 3 outlines the timetable for data collection for all performance-based and patient self-report measures.

**Table 3:** ACL study data collection timetable.

Study visit:	Enrollment	Baseline	Pre/post session	Mid-protocol (session 3)	Post-protocol (1–3 Weeks Post Intervention)
Inclusion/exclusion	X				
Consent	X				
Performance based measures					
SCAN-3		X			
HRV			X	X	X
Patient self-report measures					
SSQ-12		X		X	X
NSI		X		X	X
THI		X		X	X
PROMIS-Anxiety		X		X	X
PROMIS-Depression		X		X	X
NIH-Perceived Stress		X		X	X
SESM		X		X	X
SWLS		X		X	X
Music Therapy Symptom Assessment Form			X		
Semi Structured Interview					X

### Participants

Study participants are U.S. Military Service Members or Veterans and will be recruited from one DOD site and one VA site. Inclusion criteria specify participants must be between the ages of 18 and 65, have a confirmed diagnosis of mTBI and related sequelae, and have self-reported symptoms of auditory processing dysfunction or a formal diagnosis of CAPD. Participants will be screened using criteria from the Ohio State University TBI Identification Method (OSU TBI-ID) and self-report symptoms common in military acquired auditory processing dysfunction (5, 100). Exclusion criteria include previous participation in ACL or similar music therapy and speech-language pathology co-treatments, extreme auditory sensitivity that exacerbates existing symptoms or triggers severe adverse responses (migraines, significant emotional dysregulation), and patients with active suicidal ideation classified as high acute risk via DOD/VA clinical practice guidelines, or active psychotic symptoms not attributed to PTSD or other co-occurring medical diagnoses (46). Due to limitations in the availability of audiology services for diagnostic confirmation at each site as well as study budget, a full CAPD evaluation is not possible for every participant enrolled in this study. A total of *n* = 50 participants will be recruited from across both sites. Since the main study aims are feasibility and acceptability, a sample size calculation is not necessary. However, at 80% power, and a one-sided significance level of *p* = 0.05, assuming 20% lost to follow-up, we can detect a medium effect size (*d* = 0.40) of the intervention on study outcomes listed with 50 total participants. As such, we believe the study is adequately powered to answer the study questions.

### Materials

A combination of performance-based and patient self-report measurements will be utilized. All survey instruments are open source with the exception of the SCAN-3, in which permissions will be purchased and administered by clinicians with the required credentials. All data will be collected via Research Electronic Data Capture (REDCap).

Equipment needed to facilitate ACL includes a variety of musical instruments common in music therapy practice including fretted string instruments (acoustic and electric guitar, electric bass, ukulele), piano or electronic keyboard, pitched percussion (tongue drum), non-pitched percussion (electronic drum kit, hand percussion, auxiliary instruments), portable amplification and high-quality speakers, and a computer fitted with electronic recording software for sound editing. A decibel meter such as the National Institute for Occupational Safety and Health (NIOSH) Sound Level Meter mobile application is recommended for measuring and standardizing sound volume levels within the session (101). Additional supplies for clinical facilitation may include session specific response sheets for corresponding clinical interventions, handouts for psychoeducation, and supplementary recorded music.

## Anticipated results

We anticipate that study results will inform intervention refinement and implementation in three ways. First, qualitative feedback from provider focus group participants (SLPs, MTs) will support standardization and manualization of ACL, allowing for effective implementation as well as creating a replicable protocol for possible future clinical trials. Identifying best practice informed by emerging empirical evidence is necessary for treating complex conditions such as military acquired auditory processing dysfunction. The resources created from this manualization process can be shared with clinicians across the DOD and VA, increasing available treatment options. Second, trends in symptomatology across auditory processing and other commonly reported TBI sequelae can indicate areas of possible efficacy, clarify patient characteristics that benefit most from this treatment, and identify how ACL may add value to interdisciplinary care environments. Lastly, feedback from both patient participants, providers, and focus group clinicians can assess feasibility and acceptability across various treatment and ecological variables.

Potential challenges include issues with recruiting, scheduling, selection bias for provider-level focus groups, and access to and aggregation of data from proprietary wearable HRV devices. We acknowledge that the majority of quantitative assessment tools are self-report measures and that outcomes from one treatment protocol are difficult to assess in military and veteran healthcare systems in which patients may receive multiple treatments simultaneously. Furthermore, it is also difficult to ensure post-survey completion after discharge. Each of these potential challenges has an accompanying mitigation strategy that was deemed acceptable for study purposes, such as utilizing research team members with no prior experience of ACL for focus group facilitation to mitigate potential bias and collecting clinical outcome measures across various time points for comparison. Additionally, comparing data across two similar but distinct clinical sites allows for broader generalization of study results. Furthermore, we posit that the potential benefits of this study including creating high-reward co-treatments for military-connected populations with mTBI and auditory dysfunction outweighs any logistical issues that are commonplace in the scientific community.

## Discussion

Recent literature acknowledges the impact of auditory processing on cognitive dysfunction and suggests that interdisciplinary care is paramount when treating complex comorbid injuries such as TBI and PTSD within military-connected populations (30). Therefore, a co-treatment approach including speech, sensory (music), behavioral, and neurocognitive exercises and assistive technology may be helpful (5, 10). Music therapy uses evidence-informed interventions to address biopsychosocial-spiritual goals while motivating treatment engagement (58). Music therapy is becoming increasingly recognized in DOD and VA healthcare systems for rehabilitating cognitive, affective, sensory, language, and motor dysfunctions (64, 114). Speech-language pathology continues to be a standard of care to effectively treat CAPD, speech, language, and cognition (102). Further exploration of combining music therapy and speech-language pathology for auditory and cognitive impairment is warranted.

Music therapists and speech-language pathologists have collaboratively designed the ACL clinical co-treatment program, which has been adapted at multiple DOD sites due to variance in care models, treatment duration, and patient demographics. Therefore, a three-year multi-site feasibility and acceptability study is currently underway in the DOD and VA to standardize the protocol and prepare for a larger scale clinical trial to determine efficacy. This initial feasibility and acceptability study will glean valuable insight that can inform rigorous study design and methodology. Future clinical trials may include randomization with comparison groups to potentially include treatment as usual (standard interventions for mTBI and CAPD), music therapy only, speech language pathology only, or ACL adapted for virtual facilitation through telehealth. In addition, knowledge generated can support ongoing clinical implementation and program development, improving resources available to clinicians who support service members and veterans with auditory processing dysfunction, while adding precision to care delivery by identifying patient characteristics (symptom profiles, co-occurring diagnoses) that would most benefit from this treatment. To the authors’ collective knowledge, this study is the first to explore the impact and outcomes of a standardized music therapy and speech language pathology co-treatment protocol for military-connected populations with a diagnosis of mTBI and CAPD or other auditory processing dysfunction.

## Conclusion

This paper described a music therapy and speech language pathology co-treatment group program that treats auditory and cognition issues in military-connected populations with TBI. In order to demonstrate feasibility and acceptability within federal healthcare systems that serve military connected populations, standardization and protocolization of ACL will allow for foundational research. Further scientific inquiry will advance the understanding of ACL and its outcomes for the cognitive and psychological functioning of military-connected patients in several ways. First, determining the effectiveness of co-treatment protocols, such as ACL, provides insight into mechanisms of action in music therapy interventions and target outcomes informing future co-treatment, research questions, and study designs. Second, future studies may have a direct impact on clinical care by identifying which patient characteristics may benefit most from similar treatments. Third, creating manualized and replicable treatment protocols is necessary for expanding effective interventions for military-connected populations given the prevalence of TBI, PTSD, and related symptoms such as auditory deficits. There is a need for further development and study of this treatment, including protocol standardization, examining feasibility across military and veteran healthcare systems, demonstrating replicability, and identifying and measuring outcomes that support implementation.

## Data Availability

The original contributions presented in the study are included in the article/supplementary material, further inquiries can be directed to the corresponding author.

## Author disclaimer

The opinions and assertions expressed herein are those of the authors and do not necessarily reflect the official policy or position of the Uniformed Services University, the Defense Health Agency, the Department of Defense, nor the U.S Government. The opinions and assertions expressed herein are those of the authors and do not necessarily reflect the official policy or position of the Henry M. Jackson Foundation for the Advancement of Military Medicine, Inc., or the National Endowment for the Arts. Mention of trade names, commercial products, or organizations does not imply endorsement by the U.S. Government.

## Glossary

ACL: Auditory Cognition Lab
APT: Auditory Perception Training
ASHA: American Speech-Language-Hearing Association
CAPD: Central Auditory Processing Disorder
CDC: Center for Disease Control and Prevention
CF: Creative Forces
DOD: Department of Defense
HHIA: Hearing Handicap Inventory in Adults
HRV: Heart Rate Variability
MACT: Music Attention Control Training
MEFT: Music Executive Function Training
MT-BC: Music Therapist
NEA: National Endowment for the Arts
NIH: National Institutes of Health
NIOSH: National Institute for Occupational Safety and Health
NMT: Neurologic Music Therapy
NSI: Neurobehavioral Symptom Inventory
OSU TBI-ID: Ohio State University TBI Identification Method
PROMIS: Patient Reported Outcomes Measurement Information System
PTSD: Post Traumatic Stress Disorder
QuickSIN: Quick Speech in Noise
REDCap: Research Electronic Data Capture
SESM: Self-Efficacy for Symptom Management
SLP: Speech-Language Pathologist
SWLS: Satisfaction with Life Scale
TBI: Traumatic Brain Injury
THI: Tinnitus Handicap Inventory
U.S.: United States
VA: U.S. Department of Veterans Affairs
VHA: Veterans Health Administration
